# Seroprevalence of Anti-*Theileria equi* Antibodies in Horses from Three Geographically Distinct Areas of Romania

**DOI:** 10.3390/pathogens11060669

**Published:** 2022-06-09

**Authors:** Simona Giubega, Marius Stelian Ilie, Iasmina Luca, Tiana Florea, Cristian Dreghiciu, Ion Oprescu, Sorin Morariu, Gheorghe Dărăbuș

**Affiliations:** Department of Parasitology and Dermatology, Banat University of Agricultural Sciences and Veterinary Medicine “Regele Mihai I al Romaniei” from Timisoara, Calea Aradului 119, 300645 Timisoara, Romania; simonagiubega@gmail.com (S.G.); iasmina.luca@usab-tm.ro (I.L.); tijana.florea@usab-tm.ro (T.F.); dreghiciu.ic@gmail.com (C.D.); ioan.oprescu@fmvt.ro (I.O.); sorin.morariu@fmvt.ro (S.M.); gheorghe.darabus@fmvt.ro (G.D.)

**Keywords:** *Theileria equi*, seroprevalence, cELISA, equine piroplasmosis, Romania

## Abstract

Equine piroplasmosis (EP) is an endemic tick-borne disease found in most countries around the world. It affects all species of Equidae, and it is caused by *Theileria equi, Babesia caballi* and *T. haneyi*. The research herein is the second study on the prevalence of piroplasms in Romania conducted in the past two decades. The aim of this study was to assess the seroprevalence of anti-*Theileria equi* antibodies and the geographical distribution of this disease in the southwest, west, and northwest regions of Romania in order to obtain a more thorough understanding of the parasitological status of horses in this country. This study included 522 apparently healthy, mixed-breed horses from three different counties. The serum samples were analysed using the cELISA *Theileria equi* Antibody Test Kit. The overall seroprevalence rate was 12.84%. From the total number of positive horses, 13.96% were females and 11.21% were males. Based on the distribution of positive cases into age groups, the following values were obtained: 0–60 months: 16.26%, 60–180 months: 10.03%, and >180 months: 15.83%. There was no statistically significant difference between samples, based on age or gender. The positivity percentage in the localities included in the study ranged from 8.33 to 100%. In the population under study, the seroprevalence rate was high, indicating a possible exposure risk in this area of Romania, which could have severe effects on equids in the case of clinical manifestations of the disease. EP represents a serious threat for equine health in Romania; therefore, close and continuous monitoring of the situation is required.

## 1. Introduction

Equine piroplasmosis (EP) is a tick-borne protozoal disease of horses, donkeys, zebras, and mules caused by the haemoprotozoan parasites *Theileria equi, Babesia caballi* [1], and *T. haneyi* (newly identified species) [2,3]. The disease affects red blood cells [1,2,3], and in the literature, it has been referred to as equine malaria, biliary equine fever, and tick-borne fever and has affected all species of Equidae [4]. It has endemic characteristics in most parts of the world, with significant clinical and economic consequences due to the necessary treatment, the decreased output of animals, and the negative impact on international trade [5,6].

*Babesia* spp. And *Theileria* spp. Are protozoan parasites, mainly transmitted by ticks. There are around 30 species of *Ixodidae* identified as potential vectors [3,7,8].

The disease is endemic in most parts of the world and constitutes a risk for the welfare of equines. Its significance resides in the economic losses caused by the negative impact on international trade, namely the transportation restrictions imposed by non-endemic countries [9,10,11,12].

Only a few countries, such as Japan, the USA, Canada, the UK, those in northern Europe, Iceland, Greenland, New Zealand, and Australia are recognised as being non-endemic. EP is a B-list disease, according to the OIE classification of transmissible diseases, and has socio-economic and/or public health impacts, bearing significance in terms of international animal and animal products trade [13]. To prevent the introduction of carrier animals into non-endemic countries, only seronegative horses are allowed to be imported [14].

Due to the non-specific clinical manifestation, unnoticeable symptoms, as well as the frequent asymptomatic evolution, the disease can pass unnoticed in most animals. From a clinical point of view, the disease may range from an asymptomatic form to a super-acute form, with clinical signs associated with severe intravascular haemolysis [9,10,15]. The obvious clinical form might be a combination of factors that can be influenced by the pathogenicity of the parasite, the animal’s nutritional and immune status, the density of the infected ticks, and the infectious dose [10].

The tick species confirmed or suspected to be vectors of the causative agents of equine piroplasmosis belong to the following genera: *Amblyomma*, *Dermacentor*, *Haemaphysalis*, *Hyalomma*, *Ixodes*, and *Rhipicephalus* [7]. A systematic review of the literature on the eco-epidemiology of EP in Europe showed that the most prevalent tick species that feed on equines are *Hyalomma marginatum* and *Ixodes ricinus*, followed by *Rhipicephalus bursa*, *Haemaphysalis punctata*, *Dermacentor reticulatus*, and to a lesser extent, *Rhipicephalus sanguineus* and *Dermacentor marginatus* [12].

Various diagnostic methods are currently used to detect the infection. Serological techniques are used to identify the chronic carriers by testing their serum for the presence of specific antibodies. Molecular techniques for the detection of *T. equi* and *B. caballi* based on species-specific polymerase chain reaction (PCR) assays, targeting the specific genes, have been developed and continue to expand [15].

A series of extensive epidemiological studies targeting EP and associated factors have been conducted on different continents over the past five decades in a continuous attempt to determine the spread of these parasites within equine populations.

The difference in prevalence among countries may be due to differences in the diagnostic methods used, the number of animals subjected to studies, the occurrence of the competent vectors, climate, and the management of tick and parasite-control programs.

To the best of the authors’ knowledge, a single study has been conducted in the past two decades concerning the prevalence of piroplasms in Romania, specifically, in the rural areas of the Danube Delta [16]. Thus, this paper is the second study to focus on privately owned horses in Romania.

The aim of this study was to assess the seroprevalence of anti-*T. equi* antibodies and the geographical distribution in the southwest, west, and northwest regions of Romania, in order to obtain a more thorough understanding of the parasitological status of horses in this country.

## 2. Results

The cELISA tests revealed that 67 out of 522 analysed serum samples from horses aged between 8 and 360 months presented anti-*T. equi* IgG antibodies, with a percent inhibition above 40%, resulting in an overall seroprevalence of 12.84%. The values of the optical densities (OD) obtained through the cELISA tests, performed on the samples from the studied equines, ranged between 0.157 and 1.533.

Six variable conditions were analysed in this seroprevalence study, including the type of equid, gender, age, breed, habitat, and lifestyle (Table 1).

**Figure 1:**
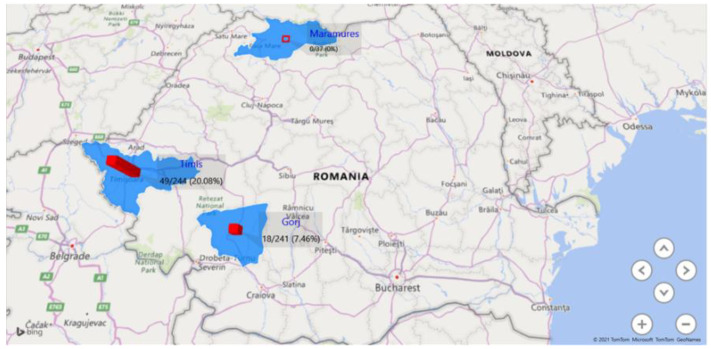
The geographical distribution of the *Theileria equi* seropositive samples.

Positive animals were detected in 14 localities from Timiș county (43.75%) and in 5 localities from Gorj county (50%). The exact number of positive samples was 49 in Timiș (20.08%) and 18 in Gorj (7.46%) (Figure 1). No positive samples were detected in localities from Maramureș county.

Of the total number of positive samples (Table 1), 43 were taken from females (13.96%) and 24 were taken from males (11.21%). Based on the age distribution, 20 samples (16.26%) belonged to the 0–60 months group, 28 samples (10.04%) belonged to the 60–180 months group, and 19 samples (15.83%) belonged to the >180 months group.

Following the data analysis, statistically significant values were noticed in terms of prevalence in each county, with emphasis on Timiș county (western Romania).

The seroprevalence of *T. equi* was significantly higher in Timiș county compared to Gorj county or Maramureș county (*p* < 0.0001 and *p* = 0.0008, respectively); however, no significant differences regarding the seroprevalence were detected between Gorj and Maramureș. There were no statistically significant differences between samples based on age or gender: the *p* value for the gender-related categories was 0.4250, and the *p* value for the age-related categories ranged from 0.0944 to 1.

## 3. Discussion

Equine piroplasmosis is a tick-borne disease that can cause serious economic losses due to its various non-specific symptoms as well as the difficulties of the field diagnosis techniques used in current veterinary practice.

So far, only one study has been conducted in Romania, focused on feral, semi-feral, and domestic horses. Therefore, the main objectives of this study were, first, to evaluate the seroprevalence rate of *T. equi* using a cELISA commercial kit and, second, to determine the possible implication of several risk factors for infection with *T. equi*. The standard serological diagnostic techniques according to the newest version of the OIE Terrestrial Manual are IFAT and cELISA, with the latter being recommended for the detection of immune responses [13]. According to certain research, IFAT discovered more suspect samples than ELISA for the diagnosis of *T. equi*, despite the latter’s excellent performance, and some samples were positive only based on ELISA and PCR, showing that their simultaneous use is beneficial [17]. The overall seroprevalence rate, as determined in this study conducted in three Romanian counties, was 12.84%, a score that is relatively low compared to other countries.

This value is lower than the European average of 30.0%, according to Nadal et al. [12], and it is also lower than the rates (38.8% seroprevalence detected via PCR) reported by Gallusova et al. in an earlier study (2010–2012) conducted on 114 domestic horses, 51 feral horses, and 13 semi-feral horses from 18 localities within or around the Danube Delta region [16].

This study reveals that *T. equi* infection is present among privately owned horses from Romania at a higher rate than expected. The high level of exposure to piroplasm parasites can be explained by the fact that animals from the rural environment do not benefit from a proper vector control program. All animals included in this study were mixed-breed, working, grazing equids from a rural area.

The seroprevalence rates obtained in this study also varied according to the geographical region. Thus, the results obtained showed that in the southwestern and western parts of Romania, the seroprevalence rates were 7.46% and 20.08%, respectively, while the results obtained from samples originating from Maramureș county, situated in northern Romania, indicated a 0% prevalence rate. This can be explained by the climatic characteristics of the Maramureș area, with Scandinavian–Baltic influences, translated through lower temperatures that are unfavourable for the evolution of competent vectors [18], and by the fact that we obtained a smaller number of samples compared to those from other counties.

Studies in Europe have led to the identification of eight tick species, belonging to five genera, that act as carriers for one or both pathogens responsible for EP: *D. reticulatus*, *D. marginatus*, *R. bursa*, *R. sanguineus*, *R. annulatus*, *H. marginatum*, *H. punctata*, and *I. ricinus*, with a prevalence of *T. equi* in ticks ranging from 0.2% to 14%. Their ability to transmit EP was suggested due to the detection of parasitic DNA in these tick species [12].

Based on the literature, 25 tick species belonging to the *Ixodidae* family have been identified so far in Romania [18], which is the species involved in EP transmission. *D. marginatus* is one of the tick vector species most commonly implicated in EP transmission [7], but this species is not cited by the literature as being present in Maramureș county, where serum samples were negative. *D. marginatus* was widespread, with an increased frequency in the western and southeastern parts, while *D. reticulatus* was found in the northeastern part of Romania. *I. ricinus* is the most widespread tick species in Romania, followed by *D. marginatus* [18,19].

Most tick species are widespread throughout the country, with a few exceptions that are distributed only in specific areas. *Dermacentor marginatus* is widespread throughout Romania, with a higher incidence in the western and southeastern areas. *D. reticulatus* is more common in the northeast; *H. punctata* is more common in the west; and *H. marginatum* is more common in the southeast, south, and west [19]. The endemic nature of the parasitic disease caused by infection with *T. equi* was also supported by the fact that most privately owned horses in Romania are autochthonous horses and not imported animals; thus, information on the prevalence of this infection within the horse populations is essential for controlling the disease and for reducing the economic losses incurred.

In accordance with our study, other researchers [20,21] found no correlation between age and *T. equi* seropositivity. Contrary to these results, other investigations revealed that older animals were more likely to be seropositive for *T. equi* [22,23,24,25,26,27,28,29,30]. Gender as a risk factor varied even more among study results, with some studies stating that females were more commonly infected, while other studies stated the opposite [26,31].

Previous studies in Europe have shown a wide range of seroprevalence rates for equine piroplasmosis: *T. equi* seroprevalence ranged from 1% in the Netherlands to 58% in France, with an estimated overall seroprevalence of 30.0% [12,32,33,34,35,36,37,38].

A number of studies concerning the seroprevalence of *T. equi* and *B. caballi* have been conducted in different countries outside Europe [15,30,39,40,41,42,43,44,45,46,47,48]. The highest rates of infection were predominantly reported in subtropical and tropical areas (in Brazil, between 80% and 90% for both parasite species) [39,40]. Mongolia, northern China, the United States [41,42], Mexico [43], Egypt [30,44,47], Chile [15] and Nigeria [45,46] have all reported cases of *T. equi* infection.

Recently, the *Theileria equi* seroprevalence rates evaluated in a study ranged from 0.9% to 100%, with a mean rate of 33.2%, representing the worldwide seroprevalence rate, calculated based on the data obtained from all studies [47].

The results obtained revealed that, in the case of horses from the southwestern and western parts of Romania, positivity for *T. equi* was noticed in at least one animal, but there were no significant differences between seropositivity and the age or gender of these animals.

The movement of horses between countries or different regions can contribute to wider spreading of new species of parasites [31,48], and understanding horse infections can help prevent this phenomenon.

None of the horses sampled in this study showed clinical signs of piroplasmosis. Once infected with *T. equi*, equines remain carriers for the rest of their lives, and this might explain why seropositivity for *T. equi* is higher [38]. Moreover, most infected horses from an endemic area are asymptomatic [49], leading to a deficient diagnosis of the *T. equi* infection.

Therefore, the results of this study correlate indirectly with other studies and show that, in Romania, competent tick vectors spread to different areas of the country [50,51] and that the parasite can be transmitted by moving the equines from one region to another, especially due to the asymptomatic nature of this disease, which makes it hard to detect clinically. Chronically infected horses can become potential reservoirs for *T. equi*; however, this status is also difficult to detect [52].

The results indicate that *T. equi* is present in the territories studied, although practicing veterinarians have not highlighted symptomatology similar to piroplasmosis, making Romania an enzootic region for the *T. equi* infection.

An overall seroprevalence rate of 12.84% demonstrates that infection with this protozoan represents a serious threat for the health of horses in Romania, and these findings may support the development of future prevention and control strategies. However, further studies, including molecular-based studies focused on the dynamics of tick transmission and the geographical distribution of vector species, are required to perform an investigation of the entire territory of this country, which, in the end, allows for better control.

## 4. Materials and Methods

### 4.1. Blood Samples and Studied Areas

This study included a total of 522 mixed-breed, apparently healthy, privately owned horses.

The horse populations from the counties included in the study were represented by animals bred in private family farms, forming groups of 8986 capita in Gorj county, 9500 capita in Timiș and 9210 capita in Maramureș [53].

The average number of horses from these three counties was 9135. The total number of horses that were sampled was determined using the epiR function of the R package [54] with a 10% expected seroprevalence, 95% confidence interval (CI), and 95% diagnostic sensitivity. The required number of horses was, thus, 31 in each county. In this study, the number of horses was increased to improve the precision of the estimated seroprevalence. The horses included in the study were selected randomly.

Data related to age, gender, and lifestyle were used to distribute the horses into the corresponding categories. The animals came from rural areas and were used as working animals.

Of the total of 522 samples, 308 were taken from females, while 214 were taken from males, aged between 8 and 360 months. Concerning the distribution into the age-related categories, a total number of 123 horses were included in the 0–60 months category, 279 were included in the 60–180 months category, and 120 were included in the >180 months category.

**Figure 2:**
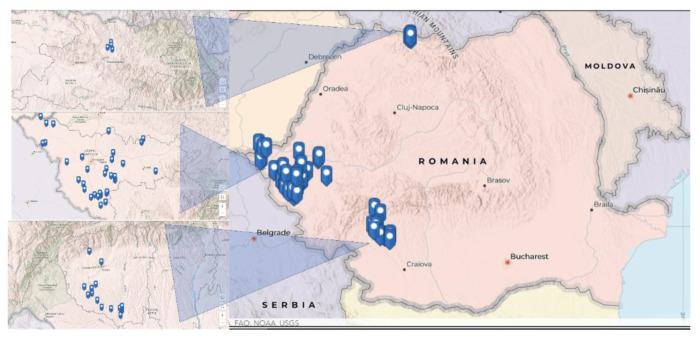
The geographical distribution of positive samples.

The blood samples were collected from the jugular vein into sterile, red top vacutainer blood collection tubes (without anticoagulant) for serologic testing, between March 2018 and June 2020, from the west, southwest, and northwest regions of Romania, namely the Gorj, Timiș, and Maramureș counties. These samples were stored at −20 °C prior to examination. Thus, 241 samples came from Gorj county (10 localities), 244 came from Timiș county (32 localities), and 37 came from Maramureș county (4 localities) (Figure 2).

### 4.2. Competitive Enzyme-Linked Immunosorbent Assay (cELISA)

The 522 serum samples were analysed using the cELISA *Teileria equi* Antibody Test Kit method (VMRD, Veterinary Medical Research and Development), according to the manufacturer’s instructions (VMRD Inc., Pullman, WA, USA). The test sensitivity was 95.0%, while the test specificity was 99.5% (data derived from the VMRD catalogue) [55]. The principle of this competitive immunosorbent assay was based on the detection of *T. equi* serum antibodies, which inhibit the binding of primary monoclonal antibodies. This primary monoclonal antibody binding to the antigen-coated plate was detected with horseradish-peroxidase (HRP)-labelled secondary antibodies. Finally, the presence of the HRP-labelled secondary antibody was quantified by the addition of an enzyme substrate and the subsequent development of a coloured product.

An absorbance microplate spectrophotometer (Sunrise, TECAN, Switzerland), with a 620/650 nm filter, was used to measure the optical density values and to record the results.

The percent inhibition (%I) was calculated according to the instructions found on the kit: % inhibition (%I) = 100 × [1 − (Sample OD ÷ NC OD)].

Test validation was performed using negative controls with optical densities >0.300 and <2.000 and positive controls, which had to show ≥40% inhibition.

Results interpretation: the samples were classified as positive if they showed ≥40% inhibition, or as negative if inhibition was <40%.

### 4.3. Statistical Analysis

The statistical analysis was conducted on two gender-related categories (males and females), three age-related categories (young animals: 0–60 months old; adults: 60–180 months old; and old animals: >180 months old) and one lifestyle-related category (working animals).

The epidemiological data related to age, gender, and activity were compared to the serological tests by applying the Fisher and Chi-squared methods (GraphPad Prism 9.2.0 (332) software). A multivariable analysis for *T. equi* seroprevalence determination was performed using Microsoft Excel. Values of *p* < 0.05 were considered significant.

## 5. Conclusions

In conclusion, the prevalence of anti-*T. equi* antibodies from the samples tested through cELISA was 12.84%. The samples were taken from horses in different regions within Romania. The positive samples were identified in 19 localities in two out of three counties included in the study, namely Timiș and Gorj. The positivity percentage in the localities included in the study ranged from 8.33% to 100%. The results indicate that *T. equi* is present in the studied territories, although practicing veterinarians have not reported the presence of any symptoms similar to piroplasmosis. EP represents a serious threat for equine health in Romania; therefore, close and continuous monitoring of the situation is required.

## Figures and Tables

**Table 1:** Distribution of positive horses according to the studied risk factors.

Epidemiological Factors	No. of Tested Equines	N * (%) *	*T. equi* OR * (95%CI *)	*p* *
	522	67 (12.84%)	0.1473 (0.1140–0.1902)	
Gender				
Female	308	43 (13.96%)	0.1623 (0.1177–0.2236)	0.425
Male	214	24 (11.21%)	0.1263 (0.0829–0.1925)	
Age group				
0–60 months	123	20 (16.26%)	0.1942 (0.1208–0.3121)	
60–180 months	279	28 (10.04%)	0.1116 (0.0757–0.1644)	
>180 months	120	19 (15.83%)	0.1881 (0.1158–0.3056)	
0–60 months vs. 60–180 months				0.0944
60–180 months vs. >180 months				0.1265
0–60 months vs. >180 months				1
Breed				
Mixed-breed	522	67 (12.84%)	0.1473 (0.1140–0.1902)	
Habitat				
Rural	522	67 (12.84%)	0.1473 (0.1140–0.1902)	
Lifestyle				
Working animals	522	67 (12.84%)	0.1473 (0.1140–0.1902)	
Counties				
Gorj	241 (5/10) **	18 (7.46%) (50%)	0.0807 (0.0502–0.1299)	
Timiș	244 (14/32) **	49 (20.08) (43.75)	0.2513 (0.1839–0.3433)	
Maramureș	37 (0/4) **	0 (0%)	0 (0–0.1038)	
Gorj vs. Timiș				<0.0001
Gorj vs. Maramureș				0.1438
Timis vs. Maramureș				0.0008

* N—number of positive samples; * **%**—prevalence; * OR—odds ratio; * CI—confidence interval; *p* *—*p* value. ** number of localities with positive samples.
